# Empowering Recovery Through Bedside Medication Accessibility

**DOI:** 10.7759/cureus.76744

**Published:** 2025-01-01

**Authors:** Ibrahim Kamel, Anu R Twayana, Deepak Kataria, Marie-Ange Alexandre, Mohamed Orabi, Luka Machavariani, Lauretta Okoye, James P Morgan

**Affiliations:** 1 Internal Medicine, St. Elizabeth's Medical Center, Boston, USA; 2 Internal Medicine, Boston Medical Center, Boston, USA; 3 Internal medicine, Texas Tech University Health Sciences Center, Odessa, USA; 4 Pharmacy, Community Care Pharmacy, Dorchester, USA; 5 Cardiology, St. Elizabeth's Medical Center, Boston, USA

**Keywords:** community teaching hospital, discharge interventions, healthcare quality improvement, hospital readmissions, medication adherence, meds-to-beds, patient satisfaction, pharmacist counseling, post-discharge care

## Abstract

Introduction

The Meds to Beds (MTB) program aims to enhance medication adherence and reduce hospital readmissions by delivering prescribed medications directly to patients' bedsides before discharge. This study evaluated the effectiveness of the MTB program in reducing 90-day readmission rates in a community teaching hospital.

Methods

This prospective study was conducted at a 159-bed community teaching hospital in Boston, MA. The study included 63 patients discharged from emergency, medical, or surgical units with at least one newly prescribed medication. Patients served as their own controls, with readmission rates compared to 90 days before and after the MTB intervention. Data were collected from electronic medical records, and paired t-tests were used to assess statistical significance, with a p-value <0.05 was used to determine statistical significance.

Principal findings

The study included 63 patients who received their medications before discharge. The mean number of admissions significantly decreased from 1.52 pre-intervention to 0.47 post-intervention (p < 0.001). Key interventions included prescription retrieval, addressing medication access barriers, and bedside delivery with personalized counseling.

Conclusions

The implementation of the MTB program significantly reduced hospital readmissions, improved medication adherence, and enhanced patient satisfaction. Addressing barriers such as transportation, high copays, and pharmacy accessibility empowered patients to adhere to prescribed regimens. This study highlights the potential benefits of expanding the MTB program to other healthcare settings to enhance care quality and reduce readmission rates.

## Introduction

The Meds to Beds (MTB) program is a patient-centered initiative designed to streamline the hospital discharge process by delivering prescribed medications directly to patients' bedsides before they leave the hospital. This service addresses common challenges that often hinder patients from obtaining their medications after discharge, such as transportation barriers, high copays, and limited pharmacy hours. By ensuring that patients are discharged with their prescribed medications in hand, the program promotes better medication adherence, reduces hospital readmissions, and enhances satisfaction among patients and their caregivers [1].

Hospital readmissions remain a pressing issue in healthcare, both in terms of frequency and financial impact. In 2018 alone, there were 3.8 million 30-day all-cause adult hospital readmissions, with an average cost of $16,300 per case [2]. While a 10.2% decrease in readmissions was observed in 2020, the challenge persists, with a readmission rate of 13.9% [2]. To address this, the Centers for Medicare and Medicaid Services (CMS) introduced penalties for hospitals with high readmission rates, encouraging healthcare systems to develop initiatives like the MTB program to improve transitions of care [1].

Implementing the MTB program involves a series of coordinated steps, including retrieving prescriptions, completing prior authorizations, resolving potential barriers to medication access, and delivering medications to the patient’s bedside along with personalized counseling [1]. This direct delivery model ensures patients not only receive their medications before leaving the hospital but also gain crucial guidance from pharmacists. Counseling sessions provide patients with clear instructions on medication use, potential side effects, and the importance of adherence, empowering them with the knowledge needed to manage their health confidently after discharge.

Research has shown mixed results in reducing hospital readmissions and improving patient outcomes. While some studies found no significant overall reduction in 30-day readmission rates [1,3], others reported benefits for specific patient populations, such as surgical patients [1] and those with low or high comorbidity burdens [4]. The program has demonstrated positive impacts on patient satisfaction [5, 6], medication adherence [7], and financial benefits for healthcare institutions [7,8]. Additionally, healthcare providers report increased workflow efficiency and a reduced administrative burden in coordinating post-discharge medications.

At its core, the MTB program emphasizes patient empowerment. By combining bedside delivery with comprehensive counseling, it ensures patients fully understand their treatment plans and feel equipped to take charge of their health. This empowerment fosters better adherence to medication regimens, encourages proactive engagement in care, and ultimately contributes to improved health outcomes.

## Materials and methods

Study design

This was a prospective quality improvement study conducted at a 159-bed community teaching hospital in Boston, Massachusetts, from September 2023 to July 2024.

Participants

Adult patients (≥18 years) discharged home with at least one newly prescribed discharge medication were eligible. Patients discharged to non-home settings (e.g., skilled nursing facilities or hospice) were excluded. Participants were identified during routine discharge planning.

Intervention

Eligible patients had their prescriptions electronically transmitted to the hospital pharmacy. Medications were delivered bedside before discharge, with pharmacists providing counseling on usage, potential side effects, and adherence strategies.

Outcomes

The primary outcome was the change in 90-day hospital readmission rates before and after the intervention.

Ethical details

This project was reviewed and determined to meet the criteria for a quality improvement initiative rather than research involving human subjects. Steward IRB issued the approval. During the project’s duration, majority authors were affiliated with Steward Healthcare which was later acquired by Boston Healthcare Center. As such, it was deemed exempt from formal IRB oversight. The primary purpose of this project was to improve internal processes and optimize patient care outcomes within the institution. All changes implemented fell within the scope of routine clinical care, posed minimal risk to patients, and adhered to standard operational procedures. Data collection was anonymized and used solely for quality improvement purposes, ensuring no additional burden or risk to patients.

Data collection and analysis

Data on demographics and readmissions were extracted retrospectively from electronic medical records. Analyses were performed using SPSS (version 29), with a paired t-test used to compare pre-and post-intervention 90-day readmission rates. Statistical significance was set at p <0.05.

## Results

Patient demographics

A total of 63 patients were included in the study, with 37 males and 26 females. The mean age for male patients was 54.3 years (SD=16.6), while for females, it was 61.65 years (SD=17.48). Ethnic distribution was as follows: African American patients (28.6%), White patients (27%), Asian patients (11.1%), Hispanic patients (9.5%), and Other (23.8%). These baseline characteristics are summarized in Table 1.

**Table 1:** Baseline characteristics.

Characteristic	Males (n=37)	Females (n=26)
Ethnicity		
African American patients	10 (27.03%)	8 (30%)
White patients	11 (29.73%)	6 (23%)
Asian patients	3 (8.11%)	4 (15.38%)
Hispanic patients	3 (8.11%)	3 (11.54%)
Other	10 (27.03%)	5 (19.23%)
Age (years)		
Mean±Standard Deviation	54.3±16.6	61.65±17.48

Readmission rates

The intervention demonstrated a statistically significant reduction in 90-day readmission rates. The mean number of hospital admissions per patient decreased from 1.52 (SD=0.90) pre-intervention to 0.47 (SD=0.82) post-intervention (p<0.001). These results are detailed in Table 2.

**Table 2:** Mean number of admissions and p-value.

Measure	Pre-intervention	Post-intervention
Mean admissions	1.52	0.47
Standard deviation	0.90	0.82
Minimum admissions	0	0
Maximum admissions	4	4
p-value	<0.001	

Program implementation outcomes

The implementation of the MTB program effectively ensured medication delivery to patients' bedsides and facilitated pharmacist counseling. Key steps included addressing barriers to medication access, completing prior authorizations, and providing personalized counseling on medication use and adherence.

## Discussion

The implementation of the MTB program in our community teaching hospital significantly reduced 90-day readmission rates, with mean admissions per patient decreasing from 1.52 to 0.47 (p<0.001). The program effectively addressed barriers to medication access by offering personalized bedside delivery and pharmacist counseling, equipping patients with the knowledge and resources needed to adhere to prescribed regimens.

Multiple studies have demonstrated the positive impact of MTB programs on readmission rates and medication adherence. Islam et al. evaluated the effect of MTB programs on 30-day readmission rates among 45,546 patients at a large community hospital. While the overall readmission rates were similar between patients receiving MTB services and those who did not (15% vs. 15.3%), subgroup analysis showed significant reductions for specific surgical populations, including females undergoing surgery (12.8% vs. 15.6%) and orthopedic/spine surgery patients (7.3% vs. 10.2%) [1].

Similarly, Stedge et al. found that MTB programs significantly improved medication adherence and reduced readmissions within 30 days by addressing common barriers to prescription fulfillment. Ensuring patients received their medications before discharge improved adherence and reduced unplanned readmissions [4]. Katz et al. demonstrated further benefits of the MTB program at Holtz Children’s Hospital, achieving an increase in the percentage of patients discharged with medications in hand (49% to 76%) and reducing prescription turnaround times to 3.5 hours. The initiative also improved caregiver satisfaction, with 96% reporting a positive experience [6].

Patel et al. expanded on these findings, showing that MTB programs at an academic medical center improved adherence and reduced readmission rates, particularly among patients with chronic conditions. Key elements of success included bedside medication delivery and pharmacist education, which supported long-term adherence [3].

Our research outcomes align closely with those of similar studies, particularly those conducted in for-profit community hospitals, such as the study by Islam et al. [1]. For-profit institutions, driven by financial and shareholder priorities, may face unique challenges in resource allocation for MTB programs. However, the consistent benefits observed across diverse settings highlight the program’s potential to improve care quality, medication adherence, and readmission outcomes [9].

Delivering quality care extends beyond discharge. Ensuring patients adhere to prescribed medication regimens is critical to maintaining the continuum of care and preventing avoidable readmissions. Our study confirms that a well-designed MTB program can achieve these goals effectively in a community teaching hospital setting.

Limitations and challenges

Although successful in reducing readmissions, the study faced operational challenges, such as pharmacy staffing during off-hours and patient compliance with payment for medications requiring a copayment. Satisfactory data were not collected, limiting insights into patient perceptions of the program.

## Conclusions

The implementation of the Meds-to-Beds program significantly reduced hospital readmissions, suggesting that such programs can effectively enhance medication adherence and patient outcomes. Future studies should explore long-term impacts and potential scalability in different hospital settings.
